# Ethyl (*E*)-3-hy­droxy-2-[(4-meth­oxy­styr­yl)­carbamo­yl]but-2-enoate

**DOI:** 10.1107/S1600536812006538

**Published:** 2012-02-24

**Authors:** Sheng-Yin Zhao, Jing Huang

**Affiliations:** a College of Chemistry, Chemical Engineering and Biotechnology, Donghua University, Shanghai 201620, People’s Republic of China

## Abstract

The title compound, C_16_H_19_NO_5_, which was synthesized from *p*-meth­oxy­cinnamic acid, has intra­molecular O—H⋯O and N—H⋯O hydrogen-bonding inter­actions. In the crystal, mol­ecules are linked by weak C—H⋯O hydrogen bonds and aromatic π–π stacking inter­actions [minimum ring centroid–centroid separation = 3.790 (1) Å].

## Related literature

For applications of 4-hy­droxy-2-pyridones, see: Jessen & Gademann (2010 ▶). For general background to the synthesis and characterization, see: Rigby & Burkhardt (1986 ▶); Rigby & Qabar (1989 ▶); Tang *et al.* (2011 ▶).
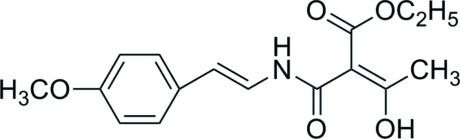


## Experimental

### 

#### Crystal data

C_16_H_19_NO_5_
*M* *_r_* = 305.32
Monoclinic, 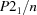

*a* = 11.1878 (15) Å
*b* = 10.5349 (14) Å
*c* = 13.5586 (18) Å
β = 101.424 (2)°
*V* = 1566.4 (4) Å^3^
*Z* = 4
Mo *K*α radiation
μ = 0.10 mm^−1^
*T* = 293 K
0.31 × 0.27 × 0.25 mm

#### Data collection

Bruker SMART CCD area-detector diffractometer
Absorption correction: multi-scan (*SADABS*; Bruker, 2003 ▶) *T*_min_ = 0.733, *T*_max_ = 1.000
8234 measured reflections
3064 independent reflections
2489 reflections with *I* > 2σ(*I*)
*R*_int_ = 0.023

#### Refinement

*R*[*F* ^2^ > 2σ(*F* ^2^)] = 0.043
*wR*(*F* ^2^) = 0.122
*S* = 1.04
3064 reflections
211 parameters
1 restraint
H atoms treated by a mixture of independent and constrained refinement
Δρ_max_ = 0.13 e Å^−3^
Δρ_min_ = −0.18 e Å^−3^

### 

Data collection: *SMART* (Bruker, 2003 ▶); cell refinement: *SAINT* (Bruker, 2003 ▶); data reduction: *SAINT*; program(s) used to solve structure: *SHELXS97* (Sheldrick, 2008 ▶); program(s) used to refine structure: *SHELXL97* (Sheldrick, 2008 ▶); molecular graphics: *SHELXTL* (Sheldrick, 2008 ▶); software used to prepare material for publication: *SHELXTL*.

## Supplementary Material

Crystal structure: contains datablock(s) I, global. DOI: 10.1107/S1600536812006538/zs2172sup1.cif

Structure factors: contains datablock(s) I. DOI: 10.1107/S1600536812006538/zs2172Isup2.hkl

Supplementary material file. DOI: 10.1107/S1600536812006538/zs2172Isup3.cml

Additional supplementary materials:  crystallographic information; 3D view; checkCIF report

## Figures and Tables

**Table 1:** Hydrogen-bond geometry (Å, °)

*D*—H⋯*A*	*D*—H	H⋯*A*	*D*⋯*A*	*D*—H⋯*A*
N1—H1*A*⋯O3	0.893 (17)	1.887 (16)	2.6088 (16)	136.7 (14)
O2—H2*A*⋯O1	0.95 (2)	1.49 (2)	2.3992 (15)	158 (2)
C1—H1⋯O3^i^	0.93	2.49	3.3533 (17)	154
C16—H16*B*⋯O5^ii^	0.96	2.59	3.411 (2)	144

Symmetry codes: (i) 
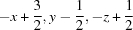
; (ii) 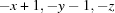.

**Fig. 1.**
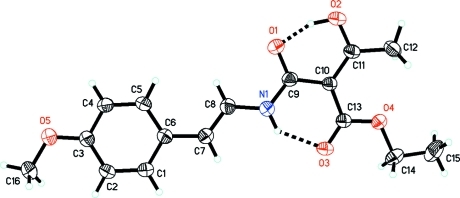
Molecular structure of the title compound showing the atom labelling scheme. Displacement ellipsoids are drawn at the 30% probability level. Hydrogen bonds are shown as dashed lines.

**Fig. 2.**
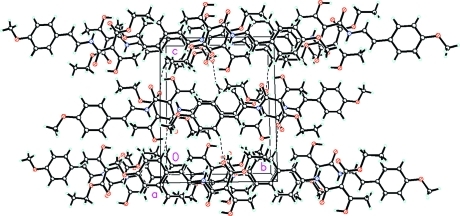
Molecular packing of the title compound viewed down the a axial direction, with intermolecular C—H···O hydrogen-bonding shown as dashed lines.

## supplementary crystallographic information

### Comment

The title compound, C_16_H_19_NO_5_ is an important intermediate for the synthesis of 4-hydroxy-2-pyridone derivatives (Jessen & Gademann, 2010). The X-ray structural analysis of this compound reported here confirms the assignment of its structure determined from spectroscopic data (Tang *et al.*, 2011). The conformation of this molecule is stabilized by intramolecular N—H···O and O—H···O hydrogen bonds (Table 1, Fig. 1). In the crystal (Fig. 2), molecules are linked by weak intermolecular C—H···O hydrogen bonds. The layers are further connected into a three-dimensional network by weak π–π stacking interactions down the *a* axiial direction of the unit cell [minimum centroid separation, 3.790 (1) Å].

### Experimental

The preparation of the title compound follows the procedure of Rigby & Burkhardt (1986) and Rigby & Qabar (1989). To an ice-cooled solution of *p*-methoxycinnamic acid (2.0 g, 11.2 mmol) in 20 ml of toluene was added triethylamine (1.6 ml, 11.6 mmol) and diphenyl phosphorazidate (DPPA, 2.9 g, 10.5 mmol). The solution was stirred at room temperature for 3 h. The acyl azide product was isolated by dilution with cold water. The organic layer was dried over anhydrous magnesium sulfate, and the solvent was removed *in vacuo* to provide a crude product. The acyl azide was dissolved in 20 ml of benzene and heated at reflux until azide decomposition was complete as monitored by TLC. The reaction mixture was then cooled to 273 K and ethyl sodioacetocaetate [prepared from ethyl acetoacetate (1.46 g, 11.2 mmol) and sodium hydride (0.37 g, 80% dispersion in oil, 12.3 mmol) in toluene (10 ml) at 273 K] was added. The reaction mixture was allowed to warm to room temperature over 2 h and was then quenched with a saturated aqueous ammonium chloride solution extracted with ether (3 times, 30 ml), rinsed with brine (3 times,30 ml), and dried over anhydrous sodium sulfate. The solvent was removed *in vacuo* to give white crystals (m.p. 367–369 K). ^1^H NMR (DMSO-d_6_, 400 MHz): 1.39 (t, 3H, CH_3_), 2.49 (s, 3H, CH_3_), 3.82 (s, 3H, CH_3_), 4.32 (q, 2H, CH_2_), 6.21 (d, 1H, =CH), 6.85 (d, 2H, Ar—H), 7.28 (d, 2H, Ar—H), 7.43 (dd, 1H, =CH), 11.04 (d, NH). Crystals suitable for X-ray diffraction were obtained by slow evaporation of a 1:1 ethyl acetate–petroleum ether solution.

### Refinement

The hydroxyl and amine H atoms were located from a difference-Fourier synthesis and their positional and isotropic displacement parameters were refined. Other H-atoms wereplaced at chemically acceptable positions and were contrained to an ideal geometry using a riding model approximation [C—H in the range 0.93–0.97 Å) and *U*_iso_(H) = 1.2 or 1.5 *U*_eq_(C)].

### Crystal data

**Table:**

C_16_H_19_NO_5_	*F*(000) = 648
*M**_r_* = 305.32	*D*_x_ = 1.295 Mg m^−^^3^
Monoclinic, *P*2_1_/*n*	Melting point = 367–369 K
Hall symbol: -P 2yn	Mo *K*α radiation, λ = 0.71073 Å
*a* = 11.1878 (15) Å	Cell parameters from 3146 reflections
*b* = 10.5349 (14) Å	θ = 4.9–54.1°
*c* = 13.5586 (18) Å	µ = 0.10 mm^−^^1^
β = 101.424 (2)°	*T* = 293 K
*V* = 1566.4 (4) Å^3^	Prismatic, yellow
*Z* = 4	0.31 × 0.27 × 0.25 mm

### Data collection

**Table:**

Bruker SMART CCD area-detector diffractometer	3064 independent reflections
Radiation source: fine-focus sealed tube	2489 reflections with *I* > 2σ(*I*)
Graphite monochromator	*R*_int_ = 0.023
φ and ω scans	θ_max_ = 26.0°, θ_min_ = 2.2°
Absorption correction: multi-scan (*SADABS*; Bruker, 2003)	*h* = −12→13
*T*_min_ = 0.733, *T*_max_ = 1.000	*k* = −12→12
8234 measured reflections	*l* = −16→10

### Refinement

**Table:**

Refinement on *F*^2^	Secondary atom site location: difference Fourier map
Least-squares matrix: full	Hydrogen site location: inferred from neighbouring sites
*R*[*F*^2^ > 2σ(*F*^2^)] = 0.043	H atoms treated by a mixture of independent and constrained refinement
*wR*(*F*^2^) = 0.122	*w* = 1/[σ^2^(*F*_o_^2^) + (0.0681*P*)^2^ + 0.143*P*] where *P* = (*F*_o_^2^ + 2*F*_c_^2^)/3
*S* = 1.04	(Δ/σ)_max_ = 0.025
3064 reflections	Δρ_max_ = 0.13 e Å^−^^3^
211 parameters	Δρ_min_ = −0.18 e Å^−^^3^
1 restraint	Extinction correction: *SHELXL97* (Sheldrick, 2008), Fc^*^=kFc[1+0.001xFc^2^λ^3^/sin(2θ)]^-1/4^
Primary atom site location: structure-invariant direct methods	Extinction coefficient: 0.100 (6)

### Special details

**Table:**

Geometry. All e.s.d.'s (except the e.s.d. in the dihedral angle between two l.s. planes) are estimated using the full covariance matrix. The cell e.s.d.'s are taken into account individually in the estimation of e.s.d.'s in distances, angles and torsion angles; correlations between e.s.d.'s in cell parameters are only used when they are defined by crystal symmetry. An approximate (isotropic) treatment of cell e.s.d.'s is used for estimating e.s.d.'s involving l.s. planes.
Refinement. Refinement of *F*^2^ against ALL reflections. The weighted *R*-factor *wR* and goodness of fit *S* are based on *F*^2^, conventional *R*-factors *R* are based on *F*, with *F* set to zero for negative *F*^2^. The threshold expression of *F*^2^ > σ(*F*^2^) is used only for calculating *R*-factors(gt) *etc*. and is not relevant to the choice of reflections for refinement. *R*-factors based on *F*^2^ are statistically about twice as large as those based on *F*, and *R*- factors based on ALL data will be even larger.

### Fractional atomic coordinates and isotropic or equivalent isotropic displacement parameters (Å^2^)

**Table:**

	*x*	*y*	*z*	*U*_iso_*/*U*_eq_	
N1	0.77258 (10)	0.36381 (11)	−0.00047 (9)	0.0537 (3)	
O1	0.76724 (11)	0.39921 (10)	−0.16424 (7)	0.0731 (3)	
O2	0.83950 (12)	0.59326 (11)	−0.22428 (7)	0.0749 (4)	
O3	0.86298 (11)	0.54370 (10)	0.12448 (7)	0.0706 (3)	
O4	0.93449 (9)	0.71806 (9)	0.06663 (7)	0.0622 (3)	
O5	0.57847 (10)	−0.35005 (9)	0.03629 (8)	0.0684 (3)	
C1	0.65197 (14)	−0.02958 (14)	0.13759 (10)	0.0588 (4)	
H1	0.6582	0.0155	0.1974	0.071*	
C2	0.61898 (13)	−0.15608 (14)	0.13560 (10)	0.0593 (4)	
H2	0.6036	−0.1950	0.1934	0.071*	
C3	0.60897 (11)	−0.22447 (13)	0.04747 (10)	0.0521 (3)	
C4	0.63126 (13)	−0.16397 (14)	−0.03769 (10)	0.0577 (4)	
H4	0.6241	−0.2091	−0.0975	0.069*	
C5	0.66365 (13)	−0.03854 (14)	−0.03493 (10)	0.0569 (4)	
H5	0.6777	0.0002	−0.0932	0.068*	
C6	0.67605 (11)	0.03238 (13)	0.05345 (9)	0.0500 (3)	
C7	0.71325 (12)	0.16556 (13)	0.06022 (11)	0.0569 (4)	
H7	0.7259	0.2023	0.1238	0.068*	
C8	0.73077 (12)	0.23922 (13)	−0.01454 (10)	0.0540 (3)	
H8	0.7145	0.2064	−0.0795	0.065*	
C9	0.79420 (12)	0.43913 (13)	−0.07438 (9)	0.0506 (3)	
C10	0.84919 (11)	0.56419 (12)	−0.05171 (8)	0.0456 (3)	
C11	0.86942 (12)	0.63708 (13)	−0.13301 (9)	0.0517 (3)	
C12	0.92260 (15)	0.76693 (15)	−0.12982 (11)	0.0666 (4)	
H12A	1.0067	0.7641	−0.0964	0.100*	
H12B	0.8785	0.8226	−0.0938	0.100*	
H12C	0.9169	0.7978	−0.1972	0.100*	
C13	0.88127 (11)	0.60534 (12)	0.05309 (9)	0.0490 (3)	
C14	0.96451 (16)	0.76631 (16)	0.16830 (12)	0.0752 (5)	
H14A	1.0243	0.7119	0.2097	0.090*	
H14B	0.8922	0.7694	0.1976	0.090*	
C15	1.01509 (19)	0.89607 (18)	0.16260 (16)	0.0984 (7)	
H15A	1.0839	0.8923	0.1302	0.148*	
H15B	1.0404	0.9296	0.2293	0.148*	
H15C	0.9536	0.9501	0.1246	0.148*	
C16	0.55675 (17)	−0.41682 (16)	0.12183 (12)	0.0745 (5)	
H16A	0.6282	−0.4132	0.1743	0.112*	
H16B	0.5379	−0.5038	0.1041	0.112*	
H16C	0.4894	−0.3787	0.1450	0.112*	
H1A	0.7911 (14)	0.3947 (15)	0.0620 (13)	0.067 (4)*	
H2A	0.8114 (17)	0.5093 (15)	−0.2163 (15)	0.099 (6)*	

### Atomic displacement parameters (Å^2^)

**Table:**

	*U*^11^	*U*^22^	*U*^33^	*U*^12^	*U*^13^	*U*^23^
N1	0.0602 (7)	0.0506 (7)	0.0500 (7)	−0.0036 (5)	0.0101 (5)	0.0008 (5)
O1	0.1040 (8)	0.0646 (7)	0.0460 (6)	−0.0161 (6)	0.0032 (5)	−0.0052 (4)
O2	0.1071 (9)	0.0762 (8)	0.0392 (5)	−0.0094 (6)	0.0096 (5)	0.0060 (5)
O3	0.1014 (8)	0.0711 (7)	0.0405 (5)	−0.0170 (6)	0.0171 (5)	0.0000 (4)
O4	0.0785 (6)	0.0580 (6)	0.0486 (5)	−0.0123 (5)	0.0089 (5)	−0.0069 (4)
O5	0.0883 (7)	0.0532 (6)	0.0650 (7)	−0.0090 (5)	0.0185 (5)	0.0023 (5)
C1	0.0702 (8)	0.0624 (9)	0.0449 (7)	−0.0065 (7)	0.0137 (6)	−0.0031 (6)
C2	0.0698 (9)	0.0625 (9)	0.0477 (7)	−0.0058 (7)	0.0169 (6)	0.0076 (6)
C3	0.0503 (7)	0.0510 (8)	0.0547 (8)	−0.0002 (5)	0.0099 (6)	0.0040 (6)
C4	0.0700 (8)	0.0582 (9)	0.0457 (7)	0.0000 (7)	0.0131 (6)	−0.0020 (6)
C5	0.0687 (8)	0.0577 (9)	0.0464 (7)	−0.0018 (6)	0.0168 (6)	0.0073 (6)
C6	0.0472 (7)	0.0536 (8)	0.0491 (7)	0.0006 (5)	0.0092 (5)	0.0031 (5)
C7	0.0604 (8)	0.0574 (8)	0.0531 (8)	−0.0051 (6)	0.0115 (6)	−0.0020 (6)
C8	0.0543 (7)	0.0502 (8)	0.0563 (8)	0.0001 (6)	0.0081 (6)	0.0002 (6)
C9	0.0531 (7)	0.0535 (8)	0.0434 (7)	0.0022 (6)	0.0054 (5)	0.0013 (5)
C10	0.0468 (6)	0.0491 (7)	0.0402 (6)	0.0024 (5)	0.0066 (5)	0.0016 (5)
C11	0.0536 (7)	0.0579 (8)	0.0428 (7)	0.0051 (6)	0.0076 (5)	0.0059 (5)
C12	0.0739 (9)	0.0681 (10)	0.0573 (8)	−0.0104 (7)	0.0120 (7)	0.0154 (7)
C13	0.0504 (7)	0.0530 (8)	0.0432 (7)	0.0020 (6)	0.0086 (5)	0.0009 (5)
C14	0.0862 (11)	0.0800 (11)	0.0569 (9)	−0.0040 (9)	0.0079 (8)	−0.0217 (8)
C15	0.0945 (13)	0.0916 (14)	0.1113 (15)	−0.0234 (11)	0.0256 (12)	−0.0477 (12)
C16	0.0902 (11)	0.0601 (10)	0.0751 (10)	−0.0113 (8)	0.0209 (9)	0.0118 (7)

### Geometric parameters (Å, º)

**Table:**

N1—C9	1.3373 (16)	C6—C7	1.4611 (19)
N1—C8	1.3938 (18)	C7—C8	1.3217 (19)
N1—H1A	0.893 (17)	C7—H7	0.9300
O1—C9	1.2676 (16)	C8—H8	0.9300
O2—C11	1.3010 (16)	C9—C10	1.4607 (18)
O2—H2A	0.952 (15)	C10—C11	1.3981 (17)
O3—C13	1.2162 (15)	C10—C13	1.4608 (17)
O4—C13	1.3247 (16)	C11—C12	1.489 (2)
O4—C14	1.4451 (17)	C12—H12A	0.9600
O5—C3	1.3672 (16)	C12—H12B	0.9600
O5—C16	1.4180 (17)	C12—H12C	0.9600
C1—C2	1.382 (2)	C14—C15	1.487 (2)
C1—C6	1.3864 (18)	C14—H14A	0.9700
C1—H1	0.9300	C14—H14B	0.9700
C2—C3	1.3808 (19)	C15—H15A	0.9600
C2—H2	0.9300	C15—H15B	0.9600
C3—C4	1.3841 (18)	C15—H15C	0.9600
C4—C5	1.369 (2)	C16—H16A	0.9600
C4—H4	0.9300	C16—H16B	0.9600
C5—C6	1.3955 (19)	C16—H16C	0.9600
C5—H5	0.9300		
			
C9—N1—C8	124.18 (12)	C11—C10—C13	123.84 (12)
C9—N1—H1A	116.9 (10)	C11—C10—C9	117.17 (11)
C8—N1—H1A	118.8 (10)	C13—C10—C9	118.98 (11)
C11—O2—H2A	104.5 (12)	O2—C11—C10	120.25 (13)
C13—O4—C14	117.43 (11)	O2—C11—C12	112.31 (12)
C3—O5—C16	117.96 (11)	C10—C11—C12	127.44 (12)
C2—C1—C6	122.19 (13)	C11—C12—H12A	109.5
C2—C1—H1	118.9	C11—C12—H12B	109.5
C6—C1—H1	118.9	H12A—C12—H12B	109.5
C3—C2—C1	119.72 (12)	C11—C12—H12C	109.5
C3—C2—H2	120.1	H12A—C12—H12C	109.5
C1—C2—H2	120.1	H12B—C12—H12C	109.5
O5—C3—C2	125.21 (12)	O3—C13—O4	120.68 (12)
O5—C3—C4	115.81 (12)	O3—C13—C10	124.62 (12)
C2—C3—C4	118.98 (13)	O4—C13—C10	114.70 (11)
C5—C4—C3	120.81 (13)	O4—C14—C15	107.02 (14)
C5—C4—H4	119.6	O4—C14—H14A	110.3
C3—C4—H4	119.6	C15—C14—H14A	110.3
C4—C5—C6	121.41 (12)	O4—C14—H14B	110.3
C4—C5—H5	119.3	C15—C14—H14B	110.3
C6—C5—H5	119.3	H14A—C14—H14B	108.6
C1—C6—C5	116.88 (12)	C14—C15—H15A	109.5
C1—C6—C7	119.98 (12)	C14—C15—H15B	109.5
C5—C6—C7	123.14 (12)	H15A—C15—H15B	109.5
C8—C7—C6	126.82 (13)	C14—C15—H15C	109.5
C8—C7—H7	116.6	H15A—C15—H15C	109.5
C6—C7—H7	116.6	H15B—C15—H15C	109.5
C7—C8—N1	123.00 (13)	O5—C16—H16A	109.5
C7—C8—H8	118.5	O5—C16—H16B	109.5
N1—C8—H8	118.5	H16A—C16—H16B	109.5
O1—C9—N1	118.65 (12)	O5—C16—H16C	109.5
O1—C9—C10	120.77 (11)	H16A—C16—H16C	109.5
N1—C9—C10	120.57 (11)	H16B—C16—H16C	109.5
			
C6—C1—C2—C3	−0.1 (2)	C8—N1—C9—C10	−173.98 (11)
C16—O5—C3—C2	−0.9 (2)	O1—C9—C10—C11	0.50 (19)
C16—O5—C3—C4	178.96 (13)	N1—C9—C10—C11	179.73 (12)
C1—C2—C3—O5	179.24 (13)	O1—C9—C10—C13	−178.68 (12)
C1—C2—C3—C4	−0.6 (2)	N1—C9—C10—C13	0.55 (18)
O5—C3—C4—C5	−179.38 (12)	C13—C10—C11—O2	179.25 (12)
C2—C3—C4—C5	0.5 (2)	C9—C10—C11—O2	0.11 (19)
C3—C4—C5—C6	0.4 (2)	C13—C10—C11—C12	−1.5 (2)
C2—C1—C6—C5	1.0 (2)	C9—C10—C11—C12	179.32 (12)
C2—C1—C6—C7	−178.64 (13)	C14—O4—C13—O3	−2.7 (2)
C4—C5—C6—C1	−1.1 (2)	C14—O4—C13—C10	177.80 (12)
C4—C5—C6—C7	178.50 (13)	C11—C10—C13—O3	179.10 (13)
C1—C6—C7—C8	−174.85 (14)	C9—C10—C13—O3	−1.8 (2)
C5—C6—C7—C8	5.6 (2)	C11—C10—C13—O4	−1.38 (18)
C6—C7—C8—N1	−176.51 (12)	C9—C10—C13—O4	177.74 (11)
C9—N1—C8—C7	178.10 (13)	C13—O4—C14—C15	−176.35 (13)
C8—N1—C9—O1	5.3 (2)		

### Hydrogen-bond geometry (Å, º)

**Table:**

*D*—H···*A*	*D*—H	H···*A*	*D*···*A*	*D*—H···*A*
N1—H1*A*···O3	0.893 (17)	1.887 (16)	2.6088 (16)	136.7 (14)
O2—H2*A*···O1	0.95 (2)	1.49 (2)	2.3992 (15)	158 (2)
C1—H1···O3^i^	0.93	2.49	3.3533 (17)	154
C16—H16*B*···O5^ii^	0.96	2.59	3.411 (2)	144

Symmetry codes: (i) −*x*+3/2, *y*−1/2, −*z*+1/2; (ii) −*x*+1, −*y*−1, −*z*.
